# Editorial: The Spectrum of Treg Subsets in Transplantation: Immune Regulation and Tolerance Induction

**DOI:** 10.3389/fimmu.2022.863148

**Published:** 2022-03-03

**Authors:** Mousa Mohammadnia-Afrouzi, Mohammad Mirzakhani, Mehdi Shahbazi, Iuliia Kotko, Hans-Dieter Volk

**Affiliations:** ^1^ Immunoregulation Research Center, Health Research Institute, Babol University of Medical Sciences, Babol, Iran; ^2^ Department of Immunology, School of Medicine, Babol University of Medical Sciences, Babol, Iran; ^3^ Student Research Committee, Mashhad University of Medical Sciences, Mashhad, Iran; ^4^ Department of Immunology, School of Medicine, Mashhad University of Medical Sciences, Mashhad, Iran; ^5^ Berlin Institute of Health (BIH) Center for Regenerative Therapies and Institute Medical Immunology, Berlin Institute of Health and Charité Universitätsmedizin Berlin, Berlin, Germany

**Keywords:** Tregs, transplantation, activated Tregs, memory Tregs, resting Tregs

Immune regulation that maintains immune homeostasis is an important part of the immune system. In this regard, regulatory T cells (Tregs) play crucial roles (1). Thymic Tregs (tTregs) express distinct T cell receptor (TCR) repertoires compared to effector T cells but, similar to effector T cells, also consist of naïve, central memory, and effector memory subsets, either in a resting or activated state, with a higher expression of functional regulatory molecules. Thymic Tregs are divided into 3 subpopulations, including naïve/resting Tregs (CD4^+^, CD45RA^+^, CD25^+^, and Foxp3^lo^), activated/memory Tregs (CD4^+^, CD45RA^–^, CD25^++^, and Foxp3^hi^), and non-Tregs (CD4^+^, CD45RA^–^, CD25^+^, and Foxp3^lo^) subpopulations. However, other markers such as HLA-DR and CD45RA may discriminate Tregs with high suppressive activity from other subpopulations. Activated Tregs have more suppressive activity than other Treg subpopulations, and there is a direct association between their frequencies and better allograft status (2, 3). Some studies have reported that activated Tregs are associated with operational tolerance or better allograft status (3–5). However, some other studies have reported inconsistent results. This may be due to the different study settings and strategies in determining Tregs (Aly et al.). Accordingly, in-depth analyses of Treg counts, subsets, and functionality in transplantation lead to a better understanding of their impact on the posttransplant course and allow more personalized immunosuppression treatments.

To evaluate the capacity or threshold of inflammation on the alloimmune response, Cross et al. cultured both activated endothelial cells (aECs) and highly aECs (haECs) separately with allogeneic peripheral blood mononuclear cells (PBMCs); haECs need a high inflammatory condition to be established. *In vitro* analyses showed that the co-culture of aECs and PBMCs was associated with higher activated and naïve Treg differentiations compared to the co-culture of haECs and PBMCs. This suggests that controlling inflammation early after transplantation may improve the immunoregulatory capacity of transplant patients through the increase of activated Tregs. To control inflammation early after transplantation, we need to control innate immune activation. Innate immune activation occurs within 24–48 h after transplantation, in which neutrophils and macrophages recognize endogenous danger signals (known as damage-associated molecular patterns). Consequently, innate immunity facilitates adaptive immune activation through efficient cytokine production and the increase of dendritic cell (DC) capacity to activate T cells (6).

MicroRNAs (miRNAs) are other factors that may improve the immunoregulatory capacity of transplant patients. In a seminal study, Yuan et al. showed the protective role of miR-223 in the transplantation outcome. miR-223 *via* inhibition of *IRAK1* reduces nuclear factor-κB (NF-κB) signaling, polarizes DCs into tolerogenic DCs, and reduces the expression of major histocompatibility complex class II and co-stimulatory molecules on the DC surface. Such DCs can reduce the T cell response, induce the Treg differentiation, and prolong the allograft survival time (7).

Based on the observations described above and the promising preclinical data, adoptive Treg therapy in transplant patients is a new therapeutic option to minimize conventional immunosuppression. Recent studies have reported the safety, efficacy, and feasibility of Treg therapy in transplant recipients (8–10). Treg therapy may contribute to the tapering of triple immunosuppression to low-dose tacrolimus monotherapy (8). However, immunosuppressive drugs may reduce the efficacy of Treg therapy. Thus, modified immunosuppressive drugs should be used to retain the efficacy of Treg therapy. Using a graft-*versus*-host disease model, Landwehr-Kenzel et al. showed that the administration of cyclosporine A–but not corticosteroids–along with Tregs had significant positive effects. In conclusion, the early control of inflammation and inhibition of genes involved in DC maturation (*i*.*e*., *IRAKs*) or adoptive Treg therapy (ideally with gene-edited immunosuppressive drug-resistant Tregs) can support posttransplant immunoregulation, allowing to minimize long-term immunosuppression.

## Author Contributions

All authors listed have made a substantial, direct, and intellectual contribution to the work and approved it for publication.

## Conflict of Interest

The authors declare that the research was conducted in the absence of any commercial or financial relationships that could be construed as a potential conflict of interest.

## Publisher’s Note

All claims expressed in this article are solely those of the authors and do not necessarily represent those of their affiliated organizations, or those of the publisher, the editors and the reviewers. Any product that may be evaluated in this article, or claim that may be made by its manufacturer, is not guaranteed or endorsed by the publisher.
